# Dosimetric assessment of patient‐specific breath‐hold reproducibility on liver motion for SBRT planning

**DOI:** 10.1002/acm2.12887

**Published:** 2020-04-26

**Authors:** Lan Lu, Zi Ouyang, Sara Lin, Anthony Mastroianni, Kevin L. Stephans, Ping Xia

**Affiliations:** ^1^ Department of Radiation Oncology Taussig Cancer Center Cleveland Clinic Cleveland OH USA

**Keywords:** active breathing control, intrafractional motion, liver SBRT, target coverage, treatment plan

## Abstract

**Purpose:**

To investigate the impact of breath‐hold reproducibility on liver motion using a respiratory motion management device.

**Methods:**

Forty‐four patients with hepatic tumors, treated with SBRT with breath‐hold, were randomly selected for this study. All patients underwent three consecutive computed tomography (CT) scans using active breath‐hold coordinator (ABC) with three repeated single breath‐hold during simulation. The three CT scans were labeled as ABC1‐CT, ABC2‐CT, and ABC3‐CT. Displacements of centroids of the entire livers among the three ABC‐CTs were measured as a surrogate for intrafractional motion. For each patient, two different treatment plans were prepared: (a) a clinical plan using a 5‐mm expansion of an ITV that encompassed all three GTVs from each of the three ABC‐CTs, and (b) a research plan using a 5‐mm expansion of the GTV from only ABC1‐CT to create PTV. The clinical plan acceptance criteria were that 95% of the PTV and 99% of the GTV received 100% of the prescription dose. Dosimetric endpoints were analyzed and compared for the two plans.

**Results:**

All shifts in the medial‐lateral direction (range: −3.9 to 2.0 mm) were within 5 mm while 7% of shifts in the anterior–posterior direction (range: −10.5 to 16.7 mm) and 11% of shifts in the superior–inferior direction (range: −17.0 to 8.7 mm) exceeded 5 mm. Six patients (14%) had an intrafraction motion greater than 5 mm in any direction. For these six patients, if a plan was created based on a PTV from a single CT (ex. ABC1‐CT), 5 of 12 GTVs captured from other ABC‐CTs would fail to meet the clinical acceptance criteria due to poor breath‐hold reproducibility.

**Conclusions:**

Non‐negligible intrafractional motion occurs in patients with poor breath‐hold reproducibility. To identify this subgroup of patients, acquiring three CTs with active breath‐hold during simulation is a feasible practical method.

## 1 INTRODUCTION

Several studies have been published on treating primary and metastatic liver tumors with stereotactic body radiotherapy (SBRT) over the past decade and have reported high rates of local control, acceptable toxicity, and low incidence of severe side effects.^1^, ^2^, ^3^, ^4^, ^5^ Effective and reliable treatment methods are crucial, as an increasing number of liver cancer patients are diagnosed every year.^6^ One of the challenges in liver SBRT delivery is dose and volume precision, which requires motion management to account for the effects of respiratory motion on liver and tumor position.

Several clinical strategies have been developed to manage liver respiratory motion in radiotherapy, particularly for SBRT, including abdominal compression,^7^ respiratory gating,^8^ four‐dimensional computed tomography (4DCT),^9^, ^10^ real‐time tumor tracking,^11^ and breath holding devices to control respiratory motion (e.g., ABC).^12^, ^13^, ^14^ These motion management methods directly impact the internal target volumes (ITV) and treatment planning margins. Four‐dimensional computed tomography using free breathing is widely used for passively capturing motion envelopes of the liver tumor during simulation. While 4DCT minimizes reliance on patient compliance, it may require larger planning margins, resulting in increased treatment target volumes. In contrast, using an active breath‐hold device such as ABC suspends a patient's breathing at a predetermined level. For patients able to tolerate a 15‐ to 20‐s breath‐hold, active breath‐hold devices significantly reduce treatment target volumes thereby sparing surrounding healthy liver tissue.^15^, ^16^, ^17^

Due to the larger fractional dose and longer delivery time than traditional fractionated IMRT, each liver SBRT treatment requires multiple breath holds using ABC. Potential errors exist when a breath‐hold position is not accurately reproduced. Our previous study^18^ has shown that even with the breath‐hold to manage the motion, 26% of patients in the study cohort exhibited intrafraction motion >3 mm in either left–right, anterior–posterior, or superior–inferior directions. To further understand the dosimetric consequences for patients with poor breath‐hold reproducibility and to propose a practical clinical solution, this study included a larger number of patients treated with liver SBRT using ABC and performed dosimetric analysis for patients with >5 mm intrafractional motion between the breath‐holds. The first goal of this study was to assess breath‐hold reproducibility by measuring the positions of the entire liver on three repeated single breath‐hold CTs using ABC. These measured positional variations were used as a surrogate for the intrafractional liver motion using ABC. The second goal of the study was to quantify dosimetric consequence if breath‐hold reproducibility are not measured during simulation.

## 2 MATERIALS AND METHODS

### 2.A Patients

This retrospective study included 44 randomly selected patients treated with liver SBRT between May 2010 and June 2012 from a research ethics board‐approved registry. To qualify for SBRT using ABC, the patients were required to tolerate more than a 15‐ to 20‐s breath‐hold and have liver tumors smaller than 5 cm.

### 2.B Simulation session

At simulation, patients were positioned supine in a BlueBAG™ BodyFIX® cushion (Elekta, Stockholm, Sweden) with arms above the head. Using the Elekta® Active Breathing Coordinator (ABC) device (Elekta, Stockholm, Sweden), patients first participated in a training session to practice repeated breath‐holding at more than 75–80% of maximal inhaled air volume for at least 15–20 s. The inhaled air volume and length of breath‐hold varied among patients, determined by patient comfort level. Without repositioning of the patient between the simulation scans, three CTs were obtained successively with each acquisition during an independent ABC‐gated breath‐hold to assess the reproducibility. These three CTs were obtained at three different phases, including one without contrast (also labeled as ABC1‐CT), one arterial phase scan, and one venous phase scan at subsequent breath‐holds. Intravenous contrast agent was administered to patients during simulation.

### 2.C Intrafractional motion analysis

The regions of interest (ROIs) including GTV and organs at risk (OARs) were contoured on the first simulation CT (ABC1‐CT). The liver contour on the first CT was used as a reference and then propagated to the second (ABC2) and the third (ABC3) CT images through deformable image registration. The liver ROIs in ABC2 and ABC3 CTs were created after manual modifications. Subsequently, the displacements of liver centroids among the three CTs were assessed by rigidly fusing each pair of CTs, aligning with the spine, and other bony structures without rotational shifts. These translational displacements represented the intrafractional motion among different ABC breath‐holds. Contouring, image registration, and displacement measurement were accomplished in MIM (MIM software Inc., Cleveland, OH).

### 2.D Treatment plan

In this study, treatment plans were prepared using two methods, first to generate a clinical treatment plan, and the second, to evaluate dosimetric impact of intrafractional liver motion. In the first method, a 5‐mm expansion of an ITV that included all three GTVs from all three CTs for each patient was used to generate the PTV. First, one GTV contour was created at each set of CTs. Contouring GTVs at non‐contrast CT images is typically difficult and dependent on tumor type, image quality, and the experience of the physicians. If the tumor is distinct from liver parenchyma, physicians were able to contour the GTV and then verified it through image registration by aligning the liver with one of contrast images. If the tumor is not distinctive from surrounding parenchyma, the GTV was first propagated from one of the contrast images to the non‐contrast image via image registration by aligning to the liver, and was then manually modified by physicians based on the location of the tumor and surrounding tissues. After GTV contours were generated at each ABC CTs, these three sets of CTs were registered using rigid image registration based on the spine and other surrounding bony structures, and GTVs from ABC2‐CT and ABC3‐CT were transferred to ABC1‐CT. The ITV was subsequently created to include all three GTVs, and was uniformly expanded for 5 mm to generate the PTV. The clinical plan acceptance criteria were that at least 95% of the PTV and 99% of the GTV/ITV received 100% of the prescription dose. For each patient, this was the clinical plan used for treatment delivery.

In the second method, a 5‐mm expansion of only the first GTV (from ABC1‐CT) was used to create PTV. This method simulated the scenario that only one simulation CT was acquired using ABC. The following ROIs were defined: GTV1, GTV2, and GTV3 were the original contours on each CT; PTV1 was correspondingly expanded by 5 mm only based on GTV1. For patients with large intrafractional liver motions >5 mm in any coordinate direction, a research plan was made based on contours on ABC1‐CT alone, such that at least 95% of the PTV1, and 99% of the GTV1 received 100% of the prescription dose. Both the clinical and the research plans met dose constraints for OARs. All treatment plans were prepared using the Pinnacle^3^ treatment planning system (Philips Radiation Oncology Systems, Fitchburg, WI).

### 2.E Treatment session

At our institution, patients treated with liver SBRT are generally under active breathing control (using ABC) to reduce respiratory motion, and planned to receive a total prescribed dose of 37.5 Gy in three fractions. The interval between the simulation and the first day of treatment for all SBRT patients is approximately 10 business days. Prior to the treatment, patients are immobilized with the devices used in simulation, and then positioned by aligning the external markers with the lasers. At the beginning of the first treatment session, patients briefly practice using ABC to maintain a stable breath‐hold position with the same threshold determined at simulation. Subsequently, a respiratory‐gated kV‐CBCT is obtained in a segmented fashion given a relative long CBCT scan time, requiring three to four breath‐holds; this is referred to as the “stop‐and‐go” CBCT technique. The thickness of the CBCT slices is 2.5 mm. After CBCT guided soft tissue alignment, the treatment beam is delivered as patients maintain a breath‐hold above the pre‐determined threshold position. Multiple (about 5 to 8) breath‐holds are needed to complete a single fraction of treatment. All patients included in this study were treated in the above‐described fashion.

### 2.F Plan quality analysis

The dosimetric endpoints were analyzed for the clinical and research plans. Target coverage was quantified by the volumes of PTV and all the GTVs that received the prescription dose. For patients with the largest liver motion, target coverage was compared between the clinical and research plans.

## 3 RESULTS

**Fig. 1:**
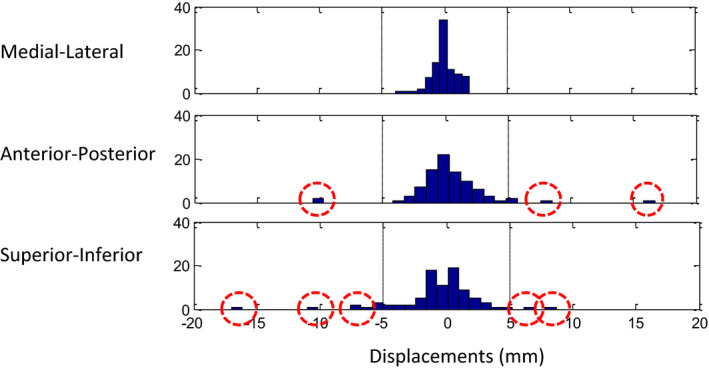
Distributions of liver centroid displacements in each direction.

The intrafractional motions were measured in two pairs of CT images for each patient. Table 1 shows the mean and range of the intrafractional motions in the medial–lateral (ML), anterior–posterior (AP), and superior–inferior (SI) directions. The mean intrafractional shift was <1 mm in the ML direction but was more than 2 mm in the SI direction with the greatest standard deviation of 2.6 mm. Six (14% of the total) patients exhibited displacements >5 mm in one direction. None of the displacements in the ML direction exceeded 5 mm, while 7% of the displacements in the AP direction and 11% of the displacements in the SI direction exceeded 5 mm. Three patients showed >10 mm shifts between breath‐holds under ABC. Figure 1 shows histograms to illustrate the distribution of the displacements in each direction. The displacements exceeding 5 mm were highlighted with red circles.

**Table 1:** Displacements of liver centroids in each direction between breath‐holds for 44 liver SBRT patients.

	Medial–lateral	Anterior–posterior	Superior–inferior
Absolute Mean ± SD	0.73 ± 0.71 mm	1.80 ± 2.51 mm	2.16 ± 2.61 mm
Min.~Max. shift	−3.94 to 2.01 mm	−10.59 to 16.69 mm	−17.01 to 8.67 mm
Shift ≥ 5 mm	0%	7%	11%

**Fig. 2:**
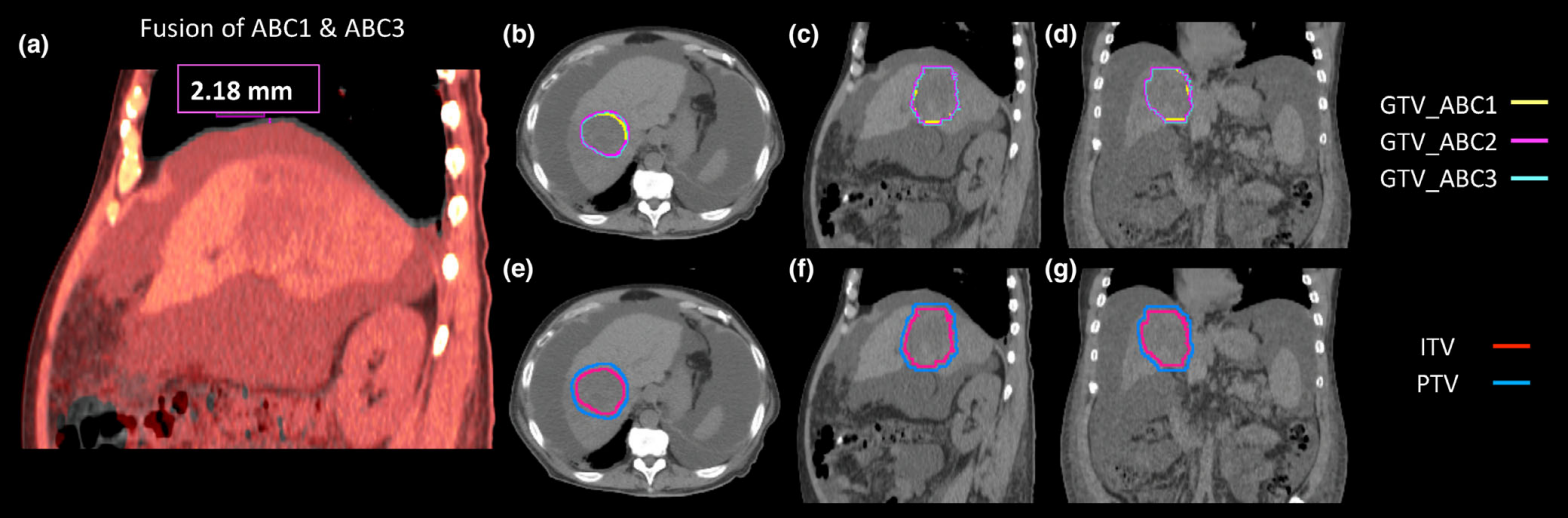
Simulation computed tomography (CT) images of a patient with good breath‐hold reproducibility. (a) Images of ABC1 and ABC3 CTs were fused. (b–d): Three GTVs contoured on each CT are shown on ABC1‐CT at axial, sagittal, and coronal views. (e–g): Internal target volumes and a PTV are shown on ABC1‐CT at axial, sagittal, and coronal views.

**Fig. 3:**
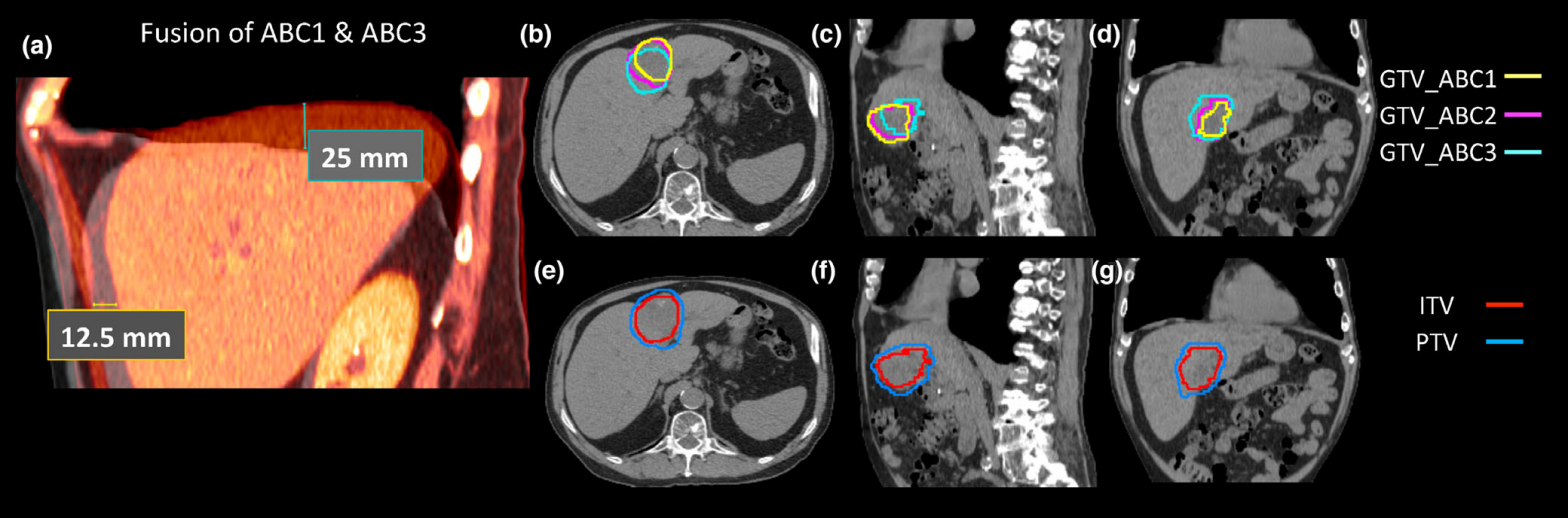
Simulation computed tomography (CT) images of a patient with poor breath‐hold reproducibility. (a) Images of ABC1 and ABC3 CTs were fused. (b–d) Three GTVs contoured on each CT are shown on ABC1‐CT at axial, sagittal and coronal views. (e–g) Internal target volumes and a PTV are shown on ABC1‐CT at axial, sagittal, and coronal views.

Figure 2 shows a representative example of a patient who was able to hold breath at approximately the same position. Three ROIs of the GTV at three consecutive simulation CTs overlapped with each other. The displacement of the liver centroid between ABC1 and ABC2 was 0.3, 2.16, and 1.10 mm in the ML, AP, and SI directions, respectively. Therefore, a 5‐mm margin used to expand the target volume to generate a PTV was sufficient to account for the intrafractional liver motion. However, a few patients were unable to reproduce breath‐holds at the same position. An extreme case (labeled as patient 1) with the largest shifts in the cohort is presented in Fig. 3. The displacements of the liver centroid for this patient were 1.3, 16.7, and 17.1 mm in the ML, AP, and SI directions, respectively. Please note that the shifts labeled on the fused CT image in Fig. 3 might differ from the centroid displacement of the liver. This is because the labeled shifts in Fig. 3 only show the displacements of the liver edges between two CTs at the selected CT slice, instead of the true 3D motion of liver centroid. As shown in Fig. 3, the GTVs from different CTs for this patient did not align well to each other.

**Fig. 4:**
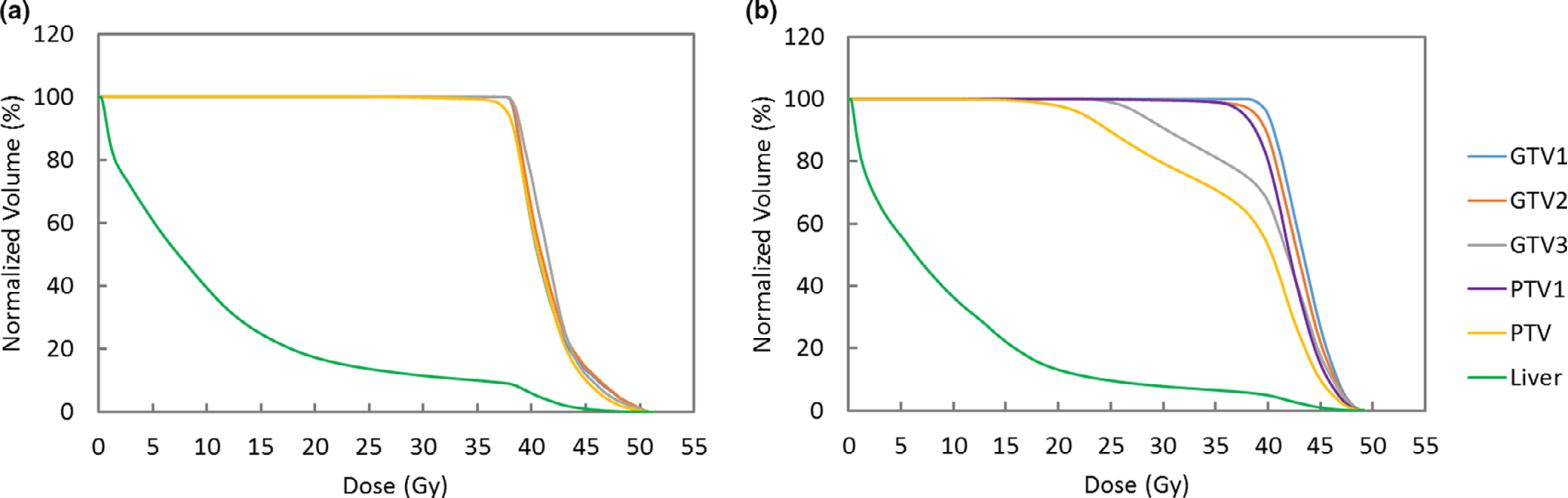
Dose‐volume histograms for patient 1 of (a) the clinical plan and (b) the research plan. In clinical plan, PTV included all the GTVs from three ABC‐CTs. In Research plan, PTV1, associated with GTV1 was used for planning. Undercoverage of GTV2, GTV3, and PTV indicates that moving targets were underdosed if motion is large.

To demonstrate plan quality differences between the clinical and research plans using two planning methods, dose volume histograms of patient 1 (the extreme case) were used as an example (Fig. 4). As shown in Fig. 4(a), the PTV and all GTVs achieved the planning goals in the clinical plan, because PTV included all the GTVs from three simulation CTs. However, in the research plan [Fig. 4(b)], since the PTV1 associated with GTV1 alone was used for treatment planning, the coverage of 100% and 95% of prescription dose only met for GTV1 and PTV1, respectively. Volumes that received 100% of the prescription dose were lower than the clinical requirements (99% for GTV) by 2% and 24% for GTV2 and GTV3, respectively. These results indicate that if the breath‐holds from patients are not reproducible, liver/tumor could move to different positions as demonstrated in the second and third ABC‐CTs in this study, leading to significantly underdosed GTVs.

**Fig. 5:**
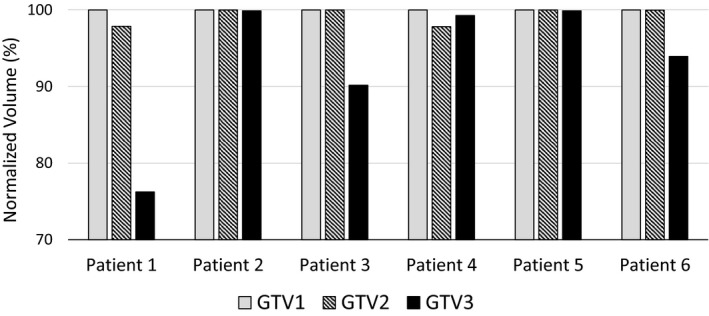
Percentage of GTV coverage by the prescription dose for six patients with large intrafractional liver motions.

The research plans were analyzed for five additional patients who had large intrafractional motions (larger than 5 mm in at least one direction). Together with patient 1 (the extreme case), 5 of 12 GTVs on ABC2 and ABC3 CTs did not meet the plan acceptance criteria (Fig. 5). To summarize, for ABC2 and ABC3 CTs, the range of GTV volumes receiving 100% prescription dose was from 76% to 100%. The average decrease in dose volume coverage was 3% for the GTVs. The clinical plans were based on the PTV that included all three GTVs, so by covering the clinical PTV, they inherently covered all three PTVs (data not shown).

## 4 DISCUSSION

To achieve highly conformal dose distributions and larger daily doses with SBRT plans requires knowledge of precise tumor position. Due to respiratory motion, it is clinically challenging to accurately localize hepatic tumors. Active breath‐hold devices have been reported to suspend the respiratory motion and reduce the treatment margin for liver radiotherapy.^12^, ^19^ However, some previous studies have indicated that liver intrafractional motion may be substantial, even with the use of ABC.^7^, ^18^ In this paper, we retrospectively studied 44 patients who were randomly selected from our clinical liver SBRT program. Instead of using population‐based planning margins for every patient, we desired to assess the individual reproducibility of breath‐holds and design patient‐specific plans correspondingly. Our results show that 86% of the patients can reproduce their liver position using ABC, but 14% of the patients were unable to maintain the same liver position during breath‐holds. The difference observed in these 14% patients may suggest that the same volume of inhaled air may not necessarily move the internal organs, such as liver, to the exact same location. The difference may also be secondary to the inherent design of this particular breath‐hold device, allowing for patient misuse. For example, some patients may breathe through the side of the mouthpiece of the ABC device.

Due to large intrafractional liver motion, dose to PTVs and GTVs may be reduced significantly, and larger planning margins will be required. Free‐breathing 4DCT is an option for patients who cannot hold breath comfortably for more than 15 s. However, the liver motion during free‐breathing would be much larger than the motion observed under active breath‐hold devices. For example, diaphragmatic displacements were found as high as 43 mm during free‐breathing in a previous report.^20^ To ensure adequate dose coverage to moving targets, a substantial volume of surrounding healthy structures needs to be encompassed in the planning target volume, with a potential increase in treatment toxicity. More than 70% of maximal inspiration is required for ABC to maintain similar lung volume, as well as internal organ positions, and excessive inter‐breath motion is not expected under ABC. Even with the ITV created based on three ABC‐CTs, the target volume is smaller than that created using a free‐breathing 4DCT. Therefore, more tissue that is normal can be spared using a breath‐hold technique. With the ITV and added margin described in this work, tumor coverage is improved for patients with large intrafractional motion. Despite the inability of a few patients to adequately hold breath and thus repeat the liver position, treatment with ABC breath‐hold is still preferred.

During simulation and treatment, patients undergo three consecutive CT with ABC, without being repositioned. In the current study, it is assumed that the liver only has translational and negligible rotational motion because these three CTs were acquired with repeated breath‐holds. Therefore, the liver deformation and rotation that mostly induced by the breathing motion were minimized. Furthermore, when assessing the intrafractional motion, the liver centroid displacement was used as a surrogate for the tumor displacement to eliminate the need for tumor delineation and the centroid is a more stable surrogate, which may not be greatly impacted by the liver deformation and rotation. Thus, we believe it is reasonable to ignore liver deformation and rotation in our study. Nevertheless, it should be caution that using liver centroid as a surrogate may introduce underestimated motion for tumors in the dome or overestimated motion in the inferior lobe.

In this study, the intrafractional liver motion was represented by three discrete CT images, while the true motion during treatment is continuous. Two potential issues exist with this approach: (a) three CTs may not be sufficient to cover the full range of the liver motion; and (b) the liver motion may differ between simulation and treatment. To confirm the intrafraction liver motion is still within the ITV range during treatments, four‐dimensional kilo‐voltage cone beam CT would be an ideal tool, or real‐time liver motion tracking is necessary.^21^, ^22^ The current results provide impetus for future studies, in which we plan to use 4D kV‐CBCT to confirm intrafraction motion prior to each treatment and to use ultrasound imaging to monitor the intrafractional liver motion during the actual SBRT treatment.

Active breath‐hold is an effective motion management technique in liver SBRT; however, non‐negligible intrafractional motion occurs in patients with poor breath‐hold reproducibility. To identify this subgroup of patients we propose a practical method of acquiring three CTs with active breath‐hold, allowing the incorporation of patient‐specific ITV in the treatment plan.

## CONFLICT OF INTERESTS

Ping Xia has received research grant from Philips, which is outside of this research topic. Other authors have no conflict of interest to declare.
